# Evaluation of the Relationship Between Oral Health and Body Mass Index

**DOI:** 10.5152/eurasianjmed.2023.23272

**Published:** 2023-10-01

**Authors:** Zeynep Betül Arslan

**Affiliations:** Department of Dentomaxillofacial Radiology, Ankara Yıldırım Beyazıt University Faculty of Dentistry, Ankara, Türkiye

**Keywords:** Boddy mass index, panoramic radiography, oral health, dental caries, obesity

## Abstract

**Objective:**

The objectives of this study is to evaluate oral health comparatively in four different groups separated according to body mass index (BMI).

**Materials and Methods:**

A total** of **352 patients who applied for different dental reasons were divided into groups according to BMI and examined radiologically. The incidence of oral health parameters (dental caries, alveolar bone loss, tooth loss, and periapical lesion) was evaluated.

**Results:**

While there was a significant difference between the groups in terms of caries, alveolar bone loss, and tooth loss (*P *< .05), there was no difference in the incidence of periapical lesions (*P *> .05).

**Conclusion:**

Obese individuals have a higher prevalence of alveolar bone loss, caries, and tooth loss. These results show that an above-normal BMI is an important factor that can negatively affect oral health.

**Main Points**

- Currently, obesity is one of the most important public health problems.
- In this study, it was determined that a BMI above normal negatively affects oral health.
- Dentists must know how nutrition affects oral health as well as how dental treatment can affect a patient’s nutritional status.

## Introduction

There are many studies investigating the relationship between oral health and general health. It is increasingly recognized that oral health is a component of overall health.^1,2^ Systemic conditions associated with oral diseases include obesity, diabetes, cardiovascular disease, lung and kidney disease, osteoporosis, pregnancy, and birth weight.^3,4^

Body mass index (BMI) is the most appropriate anthropometric measurement used for the analysis of the nutritional status of an individual.^5,6^ Body mass index is calculated by dividing weight in kilograms by height in meters squared (BMI = (Weight in kg)/(Height in m)^2^). Based on BMI, the World Health Organization (WHO) classifies adults as underweight, normal, overweight, and obese.^7^

Body mass index is the most widely used indicator of obesity.^4^ Obesity is characterized as excessive or abnormal fat accumulation that has a negative impact on health and has become one of the most important public health problems in recent years.^6,8^ Obesity has been related to many diseases, such as hypertension, diabetes, and various cancers. Although genetic predisposition is effective in the etiology of obesity, environmental factors (high-fat diet, decrease in physical activity) that have changed in recent years are also very important.^6^

In recent research, it has been reported that there is a significant relationship between BMI and oral health, and high BMI is associated with periodontitis, dental caries, and tooth loss.^6,9^ Obesity causes some changes in saliva flow. The mechanism between obesity and oral health is also based on this change. Changes in saliva flow negatively affect oral health by paving the way for oral problems such as tooth decay and tooth loss. Besides, the diet content of obese individuals is an important risk factor for dental problems.^8^

Nutrition is vital for human development and maintaining health. Dentists should know how nutrition affects general and oral health, as well as how dental treatment can affect the nutritional state of a patient. Oral health is greatly affected by daily food intake. It can also play a significant role in oral health, nutrient intake, and overall health.^10^

The aim of this study was to evaluate the relationship between body mass index and oral health in patients admitted to the faculty of dentistry.

## Materials and Methods

Ethics committee approval required for the study was obtained from Ankara Yıldırım Beyazıt University Health Sciences Ethics Committee (Number: 06-290). The study was carried out in accordance with the Declaration of Helsinki and good clinical practices.

This cross-sectional study included 352 patients over the age of 18 who applied to the oral and maxillofacial radiology clinic for various dental reasons within a 2-month period, had panoramic radiographs, and agreed to participate in the study. Informed consent forms were obtained from the participants.

Exclusion criteria: Patients under the age of 18; patients with congenital systemic diseases, musculoskeletal system diseases, immunological diseases, syndromes, pathologies that may affect the jaw and face region, any history of surgery or trauma in the head region; patients who received radiotherapy in the head and neck region; and those with poor radiographic image quality were excluded from the study. Patients with collagen tissue diseases, such as Sjögren’s syndrome, that could affect the salivary glands were also excluded.

Demographic information, such as age and gender, of the patients were recorded. Body mass index was calculated by recording the weight and height information of the individuals included in the study. According to the WHO, BMI scores were divided into 4 groups as follows:

- Underweight (<18.5 kg/m^2^).
- Normal-weight (18.5-24.9 kg/m^2^).
- Overweight (25.0-29.9 kg/m^2^).
- Obese (≥30 kg/m^2^).

In the patients included in the study, the oral health scale designed by Relvas et al^11^ and Assiri et al^3^ was modified, and the following parameters were evaluated on panoramic radiographs:

- **Caries**: None: 0; 1-3 caries: 1; 4-7 caries: 2; ≥8 caries: 3.
- **Periapical** **lesion** (except for endodontically treated teeth): None: 0; 1–2 lesion: 1; ≥3 lesion: 2.
- **Vertical** **alveolar** **bone loss**: absent: 0; present: 1.
- **Horizontal** **alveolar** **bone loss**: absent: 0; present: 1.
- **Tooth loss**: All teeth present ≥ 28: 0; <10 missing teeth: 1; >10 missing teeth: 2.
  - All third molars were excluded.

Panoramic radiographs were evaluated by an oral and maxillofacial radiology specialist with 7 years of experience. All radiographs were evaluated on the same monitor, in the same room, in ambient light. All radiographs evaluated in this study were taken on the PLANMECA ProMax instrument (Magnification Ratio 1.2) (Planmeca, Helsinki, Finland).

### Statistical Analysis

The data of the study were analyzed with the Statistical Package for the Social Sciences Statistics software, version 26 (IBM Corp., Armonk, NY, USA). For continuous variables, mean values and standard deviation were calculated; for categorical variables, percentage and frequency values were calculated. To compare categorical variables, the chi-square test was used. Statistical significance level was determined as *P *= .05.

## Results

The present study included 352 patients, with 213 women (60.51%) and 139 men (39.49%). Of these patients, 8.24% were underweight, 28.41% were normal-weight, 32.67% were overweight, and 30.68% were obese. A statistically significant difference was found between the groups according to BMI in terms of gender and mean age (*P *< .05) (Table 1).

Table 2 shows the frequency of dental caries, horizontal and vertical bone loss, tooth deficiency, and periapical lesions according to the groups. Dental caries, horizontal and vertical bone loss, and missing teeth showed statistically significant differences between the groups (*P *< .05). The incidence of periapical lesions did not differ statistically between the groups (* P* > .05). It was determined that dental caries was more common in obese individuals, and tooth loss and bone loss were more common in obese and overweight individuals.

## Discussion

Globally, obesity is one of the most important public health problems in children, adolescents, and adults. It is known that obesity is associated with many systemic diseases, but its effect on oral health is controversial.^8^ This research aimed to examine the effect of body mass index on oral health. The results of our study showed that the incidence of bone loss, dental caries, and tooth loss was higher in obese individuals. It can be thought that an increased body mass index is associated with poor oral health.

The etiology of obesity and dental caries is multifactorial. Diet is one of the most important etiological factors in both. A diet rich in carbohydrates and refined sugar is a major factor in the association between obesity and dental caries. Besides, in obese individuals, decreased stimulated salivary flow is another factor explaining its association with caries.^6,12^ In the literature, different results have been reported in studies evaluating the relationship between obesity and dental caries. Although some researchers found no association between dental caries and BMI,^6,13^ Hamasha and Willerhausen reported that overweight and obesity were significantly associated with higher caries prevalence.^14,15^ Genetic predisposition, nutritional habits, age, and lifestyle specific to societies may cause these differences.

Obesity is also accepted as a risk factor for periodontal diseases.^16^ Thomas et al^17^ found that the incidence of periodontal disease is higher in obese individuals in their cross-sectional studies. A recent review concluded that there is a significant association between obesity and periodontitis.^18^ Another research highlights that obesity can trigger alveolar bone loss.^16^ In the current study, it was determined that alveolar bone loss was significantly higher in obese individuals, which supports the literature.

In obesity, hyperplasia and hypertrophy of adipocytes cause low-grade, chronic systemic inflammation. In the case of obesity, the amount of proinflammatory cytokines tumor necrosis factor alpha, interleukin 1, and interleukin 6 produced from adipocytes increases. Tumor necrosis factor alpha is one of the first proinflammatory cytokines secreted in periodontal disease and causes the onset of periodontal disease by stimulating osteoclast formation and alveolar bone destruction. Cytokines are thought to be an important factor explaining the relationship between obesity and periodontal disease.^12,16^

Vallim et al^19^ in their study associated obesity with a high prevalence of tooth loss. As a result of the study, they emphasized that the risk of tooth loss is approximately 30% higher in obese individuals compared to normal-weight individuals over 5 years.^19^ Chari and Sabbah found that the incidence of obesity is higher in individuals with 6 or more tooth loss.^20^ Yilmaz and Efsun found that 7 or more tooth loss was more prevalent in obese groups.^8^ In this study, the presence of natural teeth, which is an important sign of oral health, was evaluated. Consistent with the literature, higher tooth loss was found in obese and overweight individuals. This relationship between obesity and tooth loss can be explained by the diet of obese individuals. A high-carbohydrate diet is thought to increase the incidence of dental caries and periodontal disease in obese individuals, resulting in a higher prevalence of tooth loss.

While Abozor et al^21^ emphasized that there was a positive relationship between periapical lesion and obesity, Yılmaz and Somay^8^ found that there was no difference between obese and normal-weight individuals in terms of periapical lesion prevalence. Similarly, it was concluded in the current investigation that BMI had no significant effect on the prevalence of periapical lesions. The common point of the 2 studies is that they were conducted in Turkish society. Individuals in the same communities may have similar dietary habits. Therefore, the effect of BMI on oral health may differ according to populations.

Nutrition is thought to be an important link between oral health and general health. Both health problems due to malnutrition and obesity are increasing all over the world.^22^ In this respect, it becomes very important to investigate the relationship between oral health and body mass index. It is thought that our study makes significant contributions to the literature with its results.

The limitation of our study is that the nutrition, oral hygiene habits, education, and socioeconomic status of the individuals are not known. More comprehensive studies with larger sample sizes in different populations are required to assess the impact of BMI on oral health.

In conclusion, obese individuals have a higher prevalence of alveolar bone loss, caries, and tooth loss. An above-normal BMI should be considered as a potential risk factor for oral health.

## Figures and Tables

**Table 1.** Demographic Characteristics of Individuals Classified According to Body Mass Index

	Underweight (n = 29)	Normal Weight (n = 100)	Overweight (n = 115)	Obese (n = 108)	*P*
Age (years) (mean ± SD)	20.86 ± 4.36	29.53 ± 11.60	38.30 ± 12.83	44.12 ± 13.79	.000^b^
Gender, n (%)					
Women	22 (75.9%)	67 (67%)	55 (47.8%)	69 (63.9%)	.005^a^
Men	7 (24.1%)	33 (33%)	60 (52.2%)	39 (36.1%)	

^a^Results of chi-square test. ^b^Results of Kruskal–Wallis test.

**Table 2.** Distribution of Oral Health Scales According to the body Mass Index Groups

		Underweight N (%)	Normal-weight N (%)	Overweight N (%)	Obese N (%)	* P *
Dental caries	0	5 (17.24)	19 (19)	15 (13.04)	10 (9.26)	.001*
	1-3	13 (44.83)	45 (45)	62 (53.91)	33 (30.56)	
	4-7	9 (31.03)	29 (29)	31 (26.96)	43 (39.81)	
	>8	2 (6.90)	7 (7)	7 (6.09)	22 (20.37)	
Horizontal bone loss	Present	1 (3.45)	23 (23)	44 (38.26)	87 (80.56)	.000*
Vertical bone loss	Present	0 (0)	5 (5)	0 (0)	35 (32.41)	.000*
Tooth loss	0	23 (79.31)	57 (57)	34 (29.57)	22 (20.37)	.000*
	1-10	6 (20.69)	41 (41)	75 (65.22)	69 (63.89)	
	>10	0 (0)	2 (2)	6 (5.22)	17 (15.74)	
Periapical lesion	0	22 (75.86)	82 (82)	87 (75.65)	74 (68.52)	.084
	1-2	7 (24.14)	18 (18)	27 (23.48)	29 (25.22)	
	>3	0 (0)	0 (0)	1 (0.87)	5 (4.63)	

* P < .005 Chi-square test.
